# Diabetic Ogilvie’s Syndrome Mimicking Colonic Volvulus: A Case Report

**DOI:** 10.7759/cureus.93813

**Published:** 2025-10-04

**Authors:** Omar Alaoui Mhammedi, Younes Oujidi, Houssam Bkiyar

**Affiliations:** 1 Department of Anesthesiology and Intensive Care, Mohammed VI University Hospital, Faculty of Medicine and Pharmacy, Oujda, MAR

**Keywords:** acute colonic pseudo-obstruction, colonic obstruction, colonic volvulus, diabetic ketoacidosis, ogilvie’s syndrome

## Abstract

Ogilvie’s syndrome, also known as acute colonic pseudo-obstruction, is a rare disorder marked by colonic dilation without any anatomical obstruction in the intestinal lumen. The dilation occurs typically in the cecum and right colon, with sometimes extension to the sigmoid colon, which can mimic a colonic volvulus, especially in geriatric patients. The involved mechanism in Ogilvie’s syndrome is an intestinal obstruction due to an alteration in the propulsion of the gastrointestinal tract content. The diagnosis of Ogilvie’s syndrome should be made only after exclusion of a specific mechanical or toxic colonic pathology. We report a case of a 73-year-old male patient with diabetes who was admitted for diabetic ketoacidosis with infectious and digestive symptoms. He later developed intestinal obstruction with significant sigmoid colon dilation. Surgery revealed colonic dilation from the cecum to the sigmoid without volvulus, but with ischemic changes. A diagnosis of diabetic Ogilvie’s syndrome mimicking sigmoid volvulus was made. The ischemic sigmoid colon was resected, and pathology confirmed no mechanical or toxic cause. The patient recovered well postoperatively.

## Introduction

Ogilvie’s syndrome, or acute colonic pseudo-obstruction, is a condition characterized by a dysfunction in the movement of gastrointestinal contents, leading to intestinal obstruction without any structural abnormalities in the intestinal lumen [1]. This dysfunction typically causes significant colonic dilation, often starting in the cecum and right colon, and may sometimes extend to the sigmoid colon and rectum [2]. In cases where Ogilvie’s syndrome is associated with marked dilation of the sigmoid colon, particularly in elderly patients, as in our reported case, the condition may mimic a colonic volvulus.

Herein, we report the case of a 73-year-old man with a history of diabetes who was initially admitted to the intensive care unit for diabetic ketoacidosis accompanied by infectious and digestive symptoms. During hospitalization, he developed an inability to pass gas or stool. Imaging revealed intestinal obstruction with marked dilation of the sigmoid colon. Surgical exploration, however, demonstrated diffuse colonic dilation extending from the cecum to the sigmoid colon, without evidence of volvulus. The sigmoid colon was notably dilated and showed ischemic changes. A diagnosis of diabetic Ogilvie’s syndrome mimicking sigmoid volvulus was established. Resection of the dilated and ischemic sigmoid colon was performed, and histopathological examination revealed no evidence of mechanical or toxic injury. The postoperative course was uneventful, and the patient recovered well.

## Case presentation

We report the case of a 73-year-old man with poorly controlled type 2 diabetes mellitus on insulin therapy. He was initially admitted to the intensive care unit for diabetic ketoacidosis, which was precipitated by an infectious syndrome characterized by fever and general weakness, along with gastrointestinal symptoms including vomiting, abdominal discomfort, and diarrhea. He denied dyspnea, cough, chest pain, headache, or dysuria and reported no medications other than insulin. On admission, his vital signs were as follows: temperature, 39.1°C; respiratory rate, 16 breaths/min; heart rate, 110 beats/min; and oxygen saturation, 100% on room air. Management consisted of intravenous hydration, insulin therapy, and empirical antibiotic treatment. The diagnosis of diabetic ketoacidosis was confirmed biologically (urinalysis showing glucose >400 mg/dL and ketones 22 mg/dL) and was thought to be secondary to a gastrointestinal infection.

On the fourth day of hospitalization, the patient developed significant abdominal distension with an inability to pass stool or flatus. Laboratory investigations revealed hypokalemia at 2.6 mmol/L (reference range: 3.5-5.0 mmol/L), which was corrected with intravenous potassium chloride. Serum sodium was 144.7 mmol/L (135-145 mmol/L), creatinine 0.94 mg/dL (0.6-1.3 mg/dL), and blood urea nitrogen 30 mg/dL (7-20 mg/dL). Glucose was 520 mg/dL (70-140 mg/dL) with an anion gap of 18 (8-16). White blood cell count was 17,000 cells/mm³ (4,000-11,000 cells/mm³), with normal hemoglobin and platelet levels. Hemoglobin A1c was 9.3% (<5.7%) (Table 1).

**Table 1:** Laboratory findings of the patient

Test	Result	Units	Normal Range
Urinalysis: glucose	>400	mg/dL	Negative
Urinalysis: ketones	22	mg/dL	Negative
Potassium (K⁺)	2.6	mmol/L	3.5 – 5.0
Sodium (Na⁺)	144.7	mmol/L	135 – 145
Creatinine	0.94	mg/dL	0.6 – 1.3
Urea nitrogen	30	mg/dL	7 – 20
Glucose (serum)	520	mg/dL	70 – 140
Anion gap	18	-	8 – 16
White blood cell count	17,000	cells/mm³	4,000 – 11,000
Hemoglobin	Normal	g/dL	~12 – 17
Platelets	Normal	/mm³	150,000 – 450,000
Hemoglobin A1c	9.3	%	<5.7

**Figure 1:**
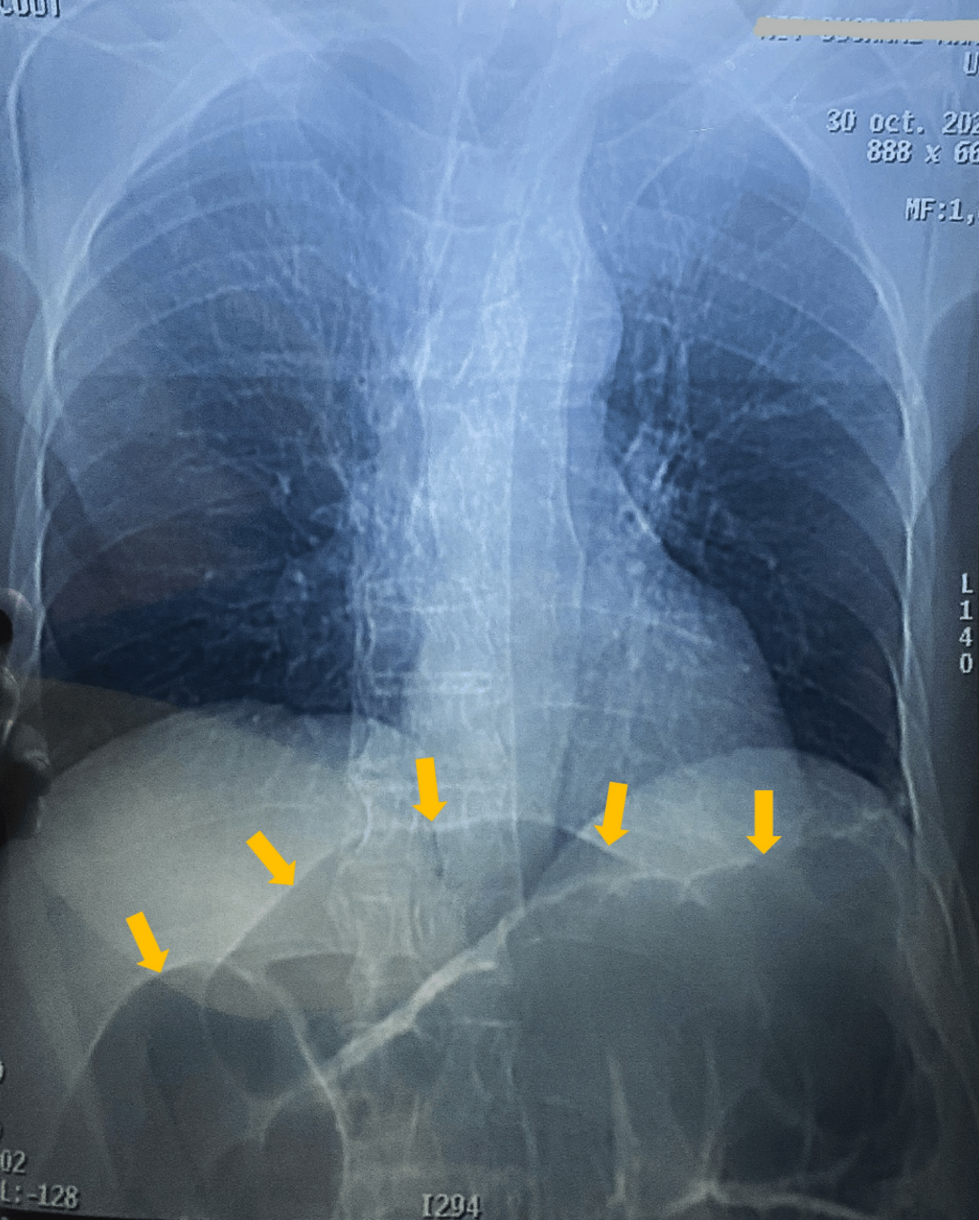
Abdominal X-ray reveals markedly distended, air-filled large bowel loops (arrows), suggestive of intestinal obstruction.

The chest radiograph was normal. An abdominal X-ray was performed and showed a dilation of the large bowel within the sigmoid colon region (Figure 1). A rapid volume resuscitation with isotonic crystalloid solution and insulin therapy was performed, associated with broad-spectrum antibiotics, although the blood culture was negative, and follow-up showed resolution of the anion gap, but with no resumption of intestinal mobility.

**Figure 2:**
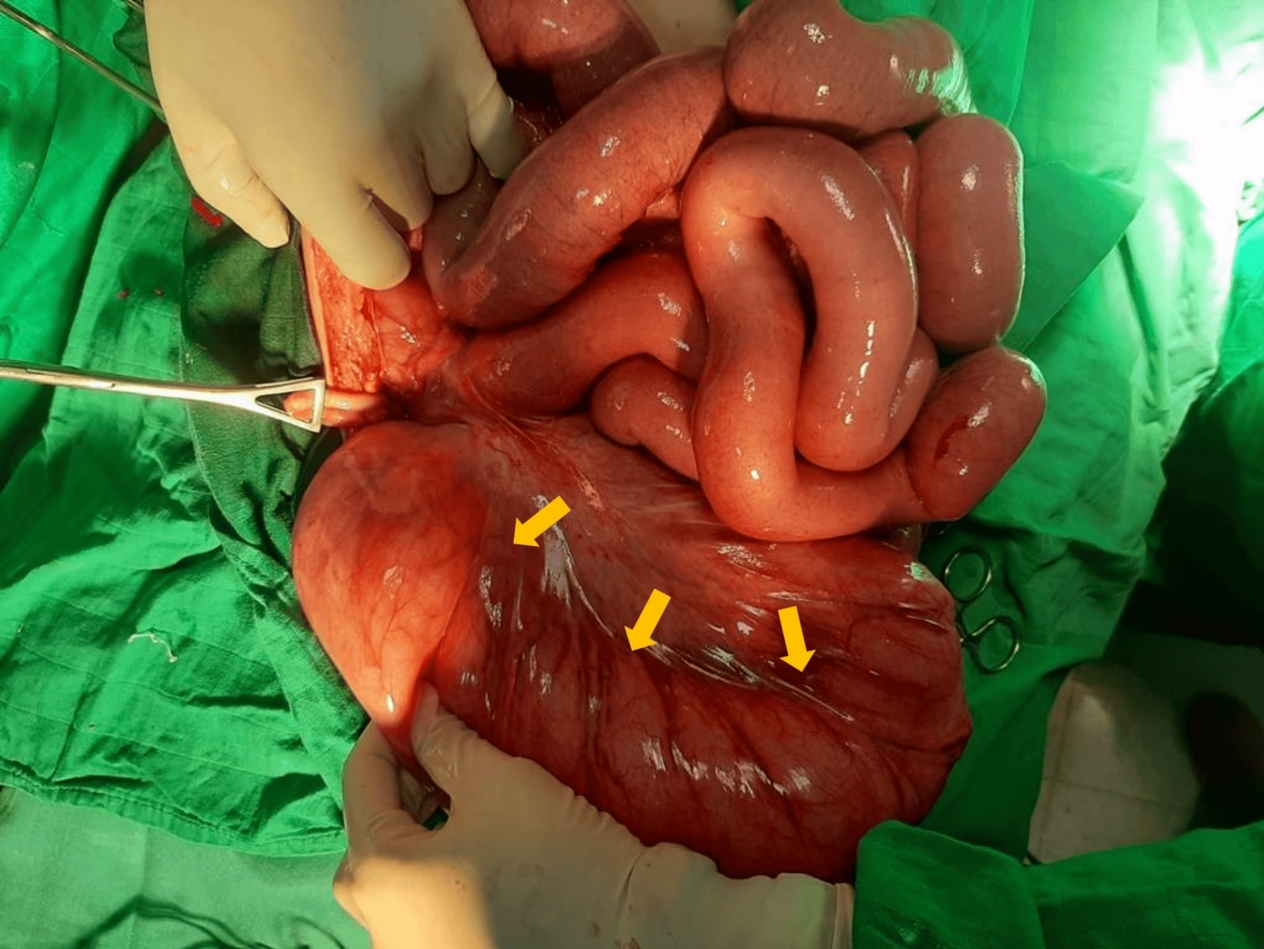
Intraoperative image shows significant colonic dilation from the cecum to the sigmoid colon (arrows), without evidence of sigmoid volvulus.

An exploratory laparotomy was performed, which revealed marked colonic dilation extending from the cecum to the sigmoid colon, without evidence of sigmoid volvulus. The dilated sigmoid segment exhibited ischemic changes (Figure 2). A diagnosis of diabetic Ogilvie’s syndrome mimicking sigmoid volvulus was established. Resection of the dilated and ischemic sigmoid colon was carried out, and pathological examination showed no evidence of mechanical obstruction or toxic injury. The postoperative course was favorable, with satisfactory recovery following resection.

## Discussion

Ogilvie’s syndrome is a rare entity where an acute dilation of the large bowel occurs in the absence of mechanical obstruction. The colonic segments typically affected by the dilation are the cecum and the right hemicolon, but can also extend to the sigmoid colon and to the rectum [3].

Ogilvie’s syndrome occurs more frequently in men, predominantly affects individuals over 60 years of age, and can be triggered by numerous risk factors, including major surgery, neurological disorders, severe illness, and the use of certain medications such as opioids, α2-adrenergic agonists, calcium channel blockers, and epidural analgesics [4]. Other associated conditions include myocardial infarction, electrolyte disturbances, malignancy, sepsis, burns, trauma, and metabolic disorders such as diabetes mellitus [5]. In our patient, the pathogenesis of Ogilvie’s syndrome was likely multifactorial, involving advanced age (73 years), diabetes mellitus, electrolyte imbalance (notably hypokalemia), and sepsis [6].

In clinical practice, Ogilvie’s syndrome must be distinguished from true colonic obstruction, including volvulus, neoplasms, strictures, paralytic ileus, and acute gastric distension [6]. When marked sigmoid dilation is present, the main differential diagnosis is colonic volvulus, which accounts for 1% to 7% of large bowel obstructions in Western Europe and the United States [7]. Delay in diagnosis and management of colonic volvulus is associated with higher mortality, which can reach 20% to 25% [8]. This risk justifies urgent surgical intervention, particularly when conservative approaches are ineffective [7]. In our case, colonic volvulus was considered the most likely differential diagnosis and therefore the primary indication for urgent laparotomy.

Although the exact pathogenesis of Ogilvie’s syndrome remains unclear, its clinical presentation closely mimics that of mechanical large-bowel obstruction [9]. For this reason, it is also referred to as acute colonic pseudo-obstruction. Patients typically present with nausea, vomiting, abdominal pain, and distension, which are indistinguishable from true colonic obstruction [10].

Management of Ogilvie’s syndrome relies on treating underlying contributing factors and decompressing the bowel. Surgical intervention may be required because of the high risk of bowel perforation [11]. This risk can be mitigated through early recognition and prompt diagnosis [11,12].

## Conclusions

Our case highlights the importance of thorough evaluation in patients with diabetic ketoacidosis, particularly in the geriatric population. Digestive symptoms in this context should not be attributed solely to concomitant gastrointestinal infection, as they may also reflect an associated Ogilvie’s syndrome. While Ogilvie’s syndrome typically presents with cecal and right colonic dilation, rare cases with sigmoid involvement can occur, closely mimicking colonic volvulus. Recognizing this diagnostic challenge is essential to ensure timely and appropriate management.
